# QMD: A new method to quantify microbial absolute abundance differences between groups

**DOI:** 10.1002/imt2.78

**Published:** 2023-01-05

**Authors:** Kai Mi, Yuyu Xu, Yiqing Li, Xingyin Liu

**Affiliations:** ^1^ State Key Laboratory of Reproductive Medicine, Center of Global Health Nanjing Medical University Nanjing China; ^2^ Department of Pathogen Biology‐Microbiology Division, Key Laboratory of Pathogen of Jiangsu Province Key Laboratory of Human Functional Genomics of Jiangsu Province Nanjing Medical University Nanjing China; ^3^ The Affiliated Suzhou Hospital of Nanjing Medical University, Suzhou Municipal Hospital, Gusu School Nanjing Medical University Nanjing China

## Abstract

A new method, quantification of microbial absolute abundance differences (QMD), was proposed to estimate the microbial absolute abundance changes of each taxon under different conditions based on the microbial relative abundance. Compared with other methods, QMD displayed greater confidence in understanding microbiome dynamics between groups. We also provide QMD software to investigate common deviations and achieve a better understanding of microbiota changes under different conditions.

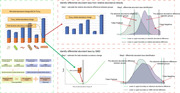

## INTRODUCTION

The development of sequencing techniques has enabled us to obtain insight into the composition of the microbiota and the dynamics of complex microbial communities. Although the absolute microbial abundance, which is the microbial load (total amount of microbes) per unit sample mass (e.g., volume or weight), is the least biased data representing the real microbiome composition, relative abundance is still commonly used in microbiome analysis. Differentially abundant taxa (DA) identification is one of the main methods of microbiome analysis that help reveal microbiome changes under different conditions. Concerns have arisen regarding DA analysis with relative abundance data or the raw count data from sequencing technologies. Comparing the raw count data of different experimental groups cannot directly describe changes in absolute microbial abundance due to the lack of sample mass normalization and amplification standardization. On the other hand, it is also difficult to interpret the compositional features using relative abundance, because increases in the relative abundance of taxa may be caused by increases in absolute microbial abundance or decreases in other taxa's absolute microbial abundance.

Two reports have shown that deviations exist between changes in relative abundance data and that in absolute abundances [1, 2]. The deviations may cause an inflated false discovery rate (FDR) in DA [3, 4]. Several DA methods have been proposed to reduce this bias effect.

Analysis of the composition of microbiomes (ANCOM) is a widely used DA tool that compares the log ratio of the abundance of each taxon to the abundance of all the remaining taxa [5], and it has been integrated into QIIME 2 [6]. The introduction of the abundance ratio has cleverly avoided gaps between relative abundance data and absolute microbial abundances. Weiss et al. concluded that ANCOM had the best performance as measured by the Mann–Whitney *U* test [7], DESeq [8], DESeq. 2 [9], edgeR [10], Voom [11], and metagenome sequencing [12] in FDR control. However, ANCOM cannot quantify microbial absolute abundance differences between groups.

The differential ranking (DR) [2] method provided evidence that the ranks of relative abundance changes are identical to the ranks of absolute microbial abundance changes. DR can quantify changes in absolute microbial abundance by constructing a multinomial regression model from relative abundance data. DR has the disadvantage that no thresholds or *p* values were provided to filter out taxa that have significantly changed, so the analysis should be limited to taxa that have changed significantly. As a result, it is suggested that the analysis should focus on taxa with very high or low changes in relative abundance.

Analysis of compositions of microbiomes with bias correction (ANCOM‐BC) [1] uses the E–M algorithm in a linear regression framework to estimate sampling fractions, which refers to the ratio of the expected raw count of a taxon in a random sample to its absolute abundance in a unit volume of the ecosystem, which implicating the performance of ANCOM‐BC might not perform well when sample sizes are very small. Using the sampling fraction for bias correction, ANCOM‐BC can infer microbial absolute abundance differences and identify differentially abundant taxa.

In this study, we propose a new method called quantification of microbial absolute abundance differences (QMD) to estimate microbial absolute abundance changes between two experimental groups based on the relative abundance data. The statistical test hypothesis for DA using QMD is also constructed, and *p* values are provided. Our results reveal that the microbial absolute abundance differences between groups of each taxon can be estimated as the relative abundance of each taxon changes plus the total microbial abundance change between groups. The total microbial abundance change (i.e., the deviation) is derived from a unary linear programming model in QMD. We validated QMD using real microbial absolute abundance change data. Compared with other methods, QMD showed better performance in microbial differential abundance quantification, DA identification, and even running speed.

## RESULTS

### QMD: Quantification of microbial differential abundances

**Figure 1:**
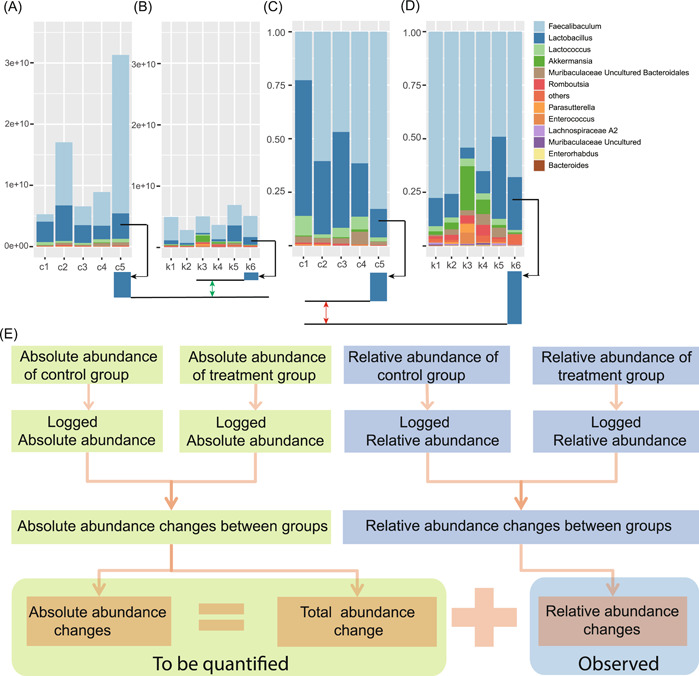
The total microbial abundance change exceeds the deviations in differentially abundant taxa identification and analysis. (A) and (B) Absolute abundance of microbiota from lower small intestines of 10‐day normal‐diet mice and ketogenic‐diet mice, respectively. (C) and (D) Relative abundance of microbiota from lower small intestines of 10‐day normal‐diet mice and ketogenic‐diet mice, respectively. The data of (A)–(D) come from [14]. It is shown the absolute abundance of Lactobacillus is decreasing while its relative abundance is increasing in ketogenic‐diet mice. (E) The relationship between relative abundance and absolute abundance in differentially abundant taxa analysis.

Previous studies showed that the microbial absolute abundance differences of bacteria are not consistent with their relative abundance differences between the two groups [13, 14]. For example, the microbial absolute abundance of *Lactobacillus* decreased in the keto group compared with the control group (Figure 1A,B), while its relative abundance increased in the keto group (Figure 1C,D).

To examine what brought out this deviation, the connections between microbial absolute abundance and relative abundance were investigated. The relationship between the microbial load of ${\mathrm{Taxa}}_{i}$ and its microbial absolute abundance can be formulated as

(1) $$A_{i,j}^{K}=m_{j}^{K}\times a_{i,j}^{K}$$

where $a_{i,j}^{K}$ represents the absolute abundance of ${\mathrm{Taxa}}_{i}$ in Group *K*, sample *j*. $m_{j}^{K}$ represents the sampling mass of Group K, sample *j*. $A_{i,j}^{K}\;$represents the microbial load of ${\mathrm{Taxa}}_{i}$ in Group *K*, sample *j*. *K* denotes the group index. For simplicity of exposition, *K* = *T*, *C*, namely the *C* (Control) group and the *T* (Treatment) Group.

Thus, the total microbial loads of all taxa in Group *K*, sample *j* can be calculated as

(2) $$\Lambda_{j}^{K}=\sum_{i}A_{i,j}^{K}=\sum_{i}m_{j}^{K}\times a_{i,j}^{K}=m_{j}^{K}\times \sum_{i}a_{i,j}^{K}$$

Denote $s_{j}^{K}$ as the sampling fraction of Group *K*, sample *j* which is defined as the ratio of the expected abundance of a taxon in a random sample to its absolute abundance in a unit volume of the eco‐system where the sample was derived from. The concept of sampling fraction is inherited from [1] and covers the sampling mass, amplicon, and sequencing operations effects on the absolute abundance. Following assumptions proposed by [1] that the sampling fractions of taxa are constant in a random sample, according to the definition of sampling fraction, we have

(3) $$A_{i,j}^{K}\times s_{j}^{K}=a_{i,j}^{K}\times m_{j}^{K}\times s_{j}^{K}=z_{i,j}^{K}$$

where $z_{i,j}^{K}$ represents the raw counts data from sequencing of ${\mathrm{Tax}a}_{i}$ in Group *K*, sample *j*.

$o_{i,j}^{K}$, the relative abundance of ${\mathrm{Taxa}}_{i}$ in Group *K*, sample *j* can be calculated from the raw count data as

(4) $$o_{i,j}^{K}=\frac{z_{i,j}^{K}}{\sum_{i}z_{i,j}^{K}}$$

Given 1–4, the connection between the relative abundance and the absolute abundance can be formulated as

(5) $$o_{i,j}^{K}=\frac{z_{i,j}^{K}}{\sum_{i}z_{i,j}^{K}}=\frac{a_{i,j}^{K}\times m_{j}^{K}\times s_{j}^{K}}{\sum_{i}a_{i,j}^{K}\times m_{j}^{K}\times s_{j}^{K}}=\frac{a_{i,j}^{K}\times m_{j}^{K}}{\sum_{i}a_{i,j}^{K}\times m_{j}^{K}}=\frac{a_{i,j}^{K}\times m_{j}^{K}}{\Lambda_{j}^{K}}$$

that is,

(6) $$a_{i,j}^{K}=\frac{o_{i,j}^{K}\times \Lambda_{j}^{K}}{m_{j}^{K}}$$

where $\frac{\Lambda_{j}^{K}}{m_{j}^{K}}$ is exactly the total microbial abundance of all taxa. Denote $\Psi_{j}^{K}=\frac{\Lambda_{j}^{K}}{m_{j}^{K}}$, we have

(7) $$a_{i,j}^{K}=\Psi_{j}^{K}\times o_{i,j}^{K}$$

Formula (7) indicates that the absolute abundance is linearly correlated with the relative abundance data. Literature has proven the correctness of Formula (7). The relative abundance data times the total microbial abundance is predictive of taxa absolute abundance. For each taxon, inferred microbial absolute abundance closely tracked the observed absolute abundance for most samples [15].

We are interested in the microbial abundance changes between groups. As we stated, each group is composed of several samples in common practices. The sample size varies from tens [14, 16] to hundreds [17, 18]. We use the difference in expectations of logged absolute abundance between groups to estimate microbial absolute abundance changes. Denote $M_{i}^{K}$ as the expectation of logged absolute abundance of ${\mathrm{Taxa}}_{i}$ in Group *K*.

Thus, the absolute abundance differences of ${\mathrm{Tax}a}_{i}$ between groups is

(8) $$∆M_{i}=M_{i}^{T}-M_{i}^{C}=\bar{\log\left(a_{i}^{T}\right)}-\bar{\log\left(a_{i}^{C}\right)}$$

To make it clear, the term *change* refers to the differences of two‐based logarithm of abundances of a taxa between groups, that is, the fold change.

Thus, we have

$${∆M}_{i}=\bar{\log\left(a_{i}^{T}\right)}-\bar{\log\left(a_{i}^{C}\right)}=\bar{\log\left(\Psi_{j}^{T}\times o_{i}^{T}\right)}-\bar{\log\left(\Psi_{j}^{C}\times o_{i}^{C}\right)}=\bar{\log\left(\Psi_{j}^{T}\right)}+\bar{\log\left(o_{i}^{T}\right)}-\left(\bar{\log\left(\Psi_{j}^{C}\right)}+\bar{\log\left(o_{i}^{C}\right)}\right)=\bar{\log\left(\Psi_{j}^{T}\right)}-\bar{\log\left(\Psi_{j}^{C}\right)}+\bar{\log\left(o_{i}^{T}\right)}-\bar{\log\left(o_{i}^{C}\right)}$$

Denote ∆Ψ as the fold change of total microbial abundance of all taxa between groups, $∆\Psi =\bar{\log\left(\Psi_{j}^{T}\right)}-\bar{\log\left(\Psi_{j}^{C}\right)}$, we have

(9) $$∆M_{i}=∆\Psi +\bar{\log\left(o_{i}^{T}\right)}-\bar{\log\left(o_{i}^{C}\right)}$$

As revealed in Formula (9), for ${\mathrm{Taxa}}_{i}$, the microbial absolute abundance differences ${∆M}_{i}$ equals the taxon's relative abundance change $\bar{\log\left((,o_{i}^{T},)\right)}-\bar{\log\left((,o_{i}^{C},)\right)}$ plus the total microbial abundance change ∆Ψ (Figure 1E). Here the total microbial abundance refers to the total microbial load per unit sample mass.

Furthermore, as the constant intercept part of the formula (9), the deviation ∆Ψ is the same for all taxa. We have noticed that to obtain the microbial absolute abundance differences for each taxon, it is only necessary to quantify the total microbial abundance change ∆Ψ because the relative abundance changes can be calculated from relative abundance data. To quantify ∆Ψ, a weak assumption that is plausible in the context of microbiome data is established: most taxa undergo relatively small microbial abundance changes. An optimization problem can be derived from this assumption, that is, minimizing the sum of absolute values of microbial abundance changes of all taxa, that is, $\sum_{{\mathrm{Taxa}}_{i}}\left|{∆M}_{i}\right|$. Given the detection rate differs between taxa, an operator, the average detection rate of ${\mathrm{Taxa}}_{i}$ in groups, $\frac{D_{i}^{T}+D_{i}^{C}}{2}$, was added as a weight to the absolute values of microbial abundance changes. The detection rate was determined as the ratio of samples with taxa detected to the total sample size. For instance, we got 100 samples and a taxon was detected in 50 samples, the corresponding detection rate was 50%. Thus, we obtain the final QMD model, that is, finding a ∆Ψ that minimizes the sum of weighted absolute values of microbial abundance changes of all taxa. This model was given in Formula (10).

(10) $$\min:\sum_{{\mathrm{Taxa}}_{i}}\frac{D_{i}^{T}+D_{i}^{C}}{2}|{∆M}_{i}|=\sum_{{\mathrm{Taxa}}_{i}}\frac{D_{i}^{T}+D_{i}^{C}}{2}\left|∆\Psi +\bar{\log\left(o_{i}^{T}\right)}-\bar{\log\left(o_{i}^{C}\right)}\right|$$

where $D_{i}^{C}$ and $D_{i}^{T}$ are the detection rates of ${\mathrm{Taxa}}_{i}$ in the control group and treatment group, respectively.

To find the optimization point in Formula (10), QMD adopted a traversal from −10 to 10 by step 0.01. This traversal scope covers an increase in microbial abundance by nearly 1000 times or a decrease to one‐thousandth compared with the control group. The traversal scope and step can be modified in the provided QMD software. After the quantification of ∆Ψ, the microbial absolute abundance differences ${∆M}_{i}$ for each ${\mathrm{Taxa}}_{i}$ can be calculated according to Formula (9).

### QMD‐based differentially abundant taxa identification

The DA analysis methods, for example, two‐sample *t*‐tests and Mann–Whitney *U* tests, have limited power to detect. Formula (9) indicates that ∆Ψ contributes to the inflated FDR of the traditional popular methods. If a positive ∆Ψ was obtained, for example, the total microbial abundance would increase twofold in the treatment group, and the observed changes in relative abundance between groups would be smaller than the changes in absolute abundances. Notably, if the relative abundance did not change in this case, the real microbial abundances would actually increase twofold, meaning that the microbial abundances in the treatment group were four times larger than that in the control group. A negative ∆Ψ creates the opposite effect.

Naturally, we constructed a new statistical test hypothesis:

$$H_{0}:\;\mathrm{The}\;\mathrm{microbial}\;\mathrm{absolute}\;\mathrm{abundance}\;\mathrm{has}\;\mathrm{not}\;\mathrm{changed}\;\mathrm{between}\;\mathrm{groups}$$

to identify differentially abundant taxa, as follows:

(11) $$H_{0}:∆M_{i}=∆\Psi +\bar{\log\left(o_{i}^{T}\right)}-\bar{\log\left(o_{i}^{C}\right)}=0$$

This hypothesis can be rewritten as:

(12) $$H_{0}:∆\Psi +\bar{\log\left(o_{i}^{T}\right)}=\bar{\log\left(o_{i}^{C}\right)}$$

Formula (12) is the proposed QMD‐based differentially abundant taxa identification method. After the quantification of ∆Ψ, the Mann–Whitney *U* test was used to conduct a statistical test. To facilitate the application of QMD, releases of QMD software were available at https://github.com/Xingyinliu-Lab/QMD/tree/master/GUI_QMD/release. We also provided a video tutorial at https://www.youtube.com/watch?v=LPti1vgoiCo. Since the test needs to be performed for all taxa, the QMD software also supports Benjamini/Hochberg tests for *p* value FDR adjustment.

### QMD validation by real data

Recent studies have focused on measuring microbial absolute abundances. For example, digital PCR [14], spike‐in [19, 20], flow cytometry [13], quantitative PCR [21], and hamPCR [22] have been adopted to make an anchor point from which to convert relative data to microbial absolute abundances. We selected four instances, LSI, STOOL, B1, and B2, from the above‐cited literature to validate our proposed QMD [13, 14]. Detailed information about the four instances is given in Supporting Information: Table S1.

**Figure 2:**
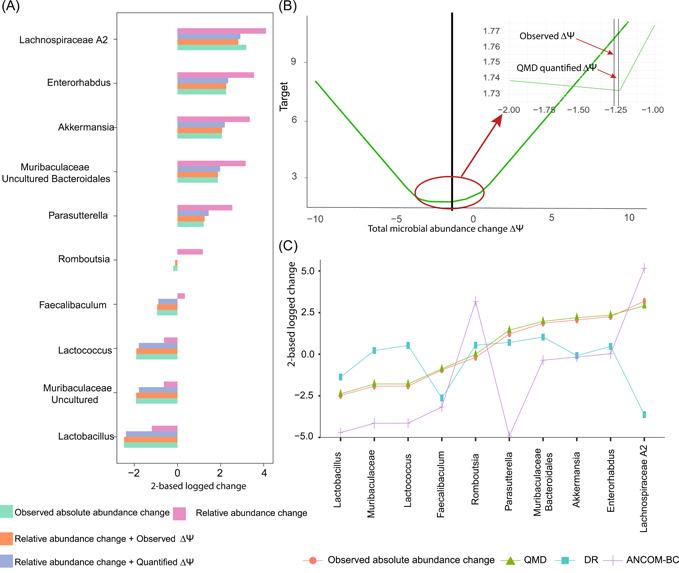
QMD validation in the LSI data set. (A) Validation of the relationship between relative abundance changes and absolute abundance changes. Either the observed or QMD‐quantified total microbial abundance change can cancel out the deviation. (B) The visualization of traversal processes to find the quantified absolute abundance differences by QMD in LSI. A magnifier was provided in the optimal target zone. (C) Performance comparison of QMD, DR, and ANCOM‐BC in absolute abundance differences quantification. ANCOM‐BC, analysis of compositions of microbiomes with bias correction; QMD, quantification of microbial absolute abundance differences.

From the measured microbial absolute abundances, the total microbial abundance change ∆Ψ can be calculated (Table 1). Clearly, ∆Ψ can perfectly correct the existing shift between relative abundance differences and absolute abundance differences between groups (Figure 2A and Supporting Information: Figure S1A). This finding validates Formula (9).

**Table 1:** Results summary for correctness validation

#	Instances	Observed ∆Ψ	Quantified ∆Ψ by QMD	Mean absolute error			False discovery rate			Proportion of differentially abundant taxa (%)
				QMD	ANCOM‐BC	DR	QMD	ANCOM	MWU	
1	LSI	−1.284	−1.250	0.144	2.708	2.020	0.200	0.600	0.300	50
2	STOOL	−1.514	−1.210	0.319	1.433	2.008	0.119	0.357	0.357	50
3	B1	−0.384	−0.180	0.593	1.440	1.300	0.250	0.250	0.125	25
4	B2	−0.869	−0.920	0.704	2.541	1.533	0.105	0.263	0.105	10

*Note*: MWU denotes the application of the Mann–Whiney *U* test directly to the relative abundance to identify differentially abundant taxa.

Abbreviations: ANCOM, Analysis of the composition of microbiomes; ANCOM‐BC, analysis of compositions of microbiomes with bias correction; DR, differential ranking; QMD, quantification of microbial absolute abundance differences.

The total microbial abundance $\hat{∆\Psi }$ was quantified using optimization traversal. As shown in Figure 2B and Supporting Information: Figure S1B, the target function in QMD is convex, and there's no local best trap. The quantified $\hat{∆\Psi }$ were close to the observed true ∆Ψ, especially in the instances of LSI and B2 (Table 1). The quantified $\hat{∆\Psi }$ plus the relative abundance changes were close to the observed absolute abundance differences (Figure 2A and Supporting Information: Figure S1A). This result validated the QMD model presented in Formula (10). Furthermore, we compared the performance of QMD, ANCOM‐BC, and DR in microbial absolute abundance differences quantification. QMD outperformed the other methods in all four instances (Figure 2C, Supporting Information: Figures S1C and S2A,B, and Table 1).

To validate the power of QMD in differentially abundant taxa identification, the FDR of QMD, ANCOM, and Mann–Whitney *U* tests were calculated for the four instances. QMD outperformed the other methods in LSI, STOOL, and B2. The Mann–Whitney *U* test performed best in the instance of B1 (Table 1).

We next compared the performance of QMD, ANCOM, and Mann–Whitney *U* tests in several human gut microbiome data sets (Supporting Information: Table S2). The total microbial abundance change indicates a different pattern in the gut microbiome of patients with different diseases (Supporting Information: Figure S3A). A decreasing tendency in total microbial abundance could be found in colorectal cancer (CRC) patients (Supporting Information: Figure S3A). This pattern might be connected to the differential host–microbiome interaction types in patients with different diseases. Consumption of antibiotics like antibiotics cocktail treatment could also affect the total microbial abundance change. For example, the total microbial abundance change in Inflammatory bowel disease (IBD) shows a relatively large variation. This suggests that careful attention should be paid when making QMD analysis crossing multistudies. The heterogeneity of the population, and the above‐mentioned antibiotics consumption history, deity, sex, age, and other microbiome‐relating factors should be considered before making data integration.

Another finding is for taxa with relatively small microbial abundance changes, the Mann–Whitney *U* test output yielded a nearly opposite result (Supporting Information: Figure S3B). ANCOM may generate false negatives for taxa with high absolute abundance changes. A possible reason is that ANCOM focuses on the abundance ratio between taxa. In most cases, more than one taxon undergoes high absolute abundance changes, and the ratio between these taxa may not be altered as greatly as the taxa abundance itself.

### QMD performance in microbial differential abundance quantification is robust

To further investigate various sample sizes, the proportions of differentially abundant taxa, and the effect of microbial abundance changes on the performance of QMD, we conducted a benchmark simulation using three data set series: H2029, Obesity, and global patterns (GP) (see Section Methods for details).

**Figure 3:**
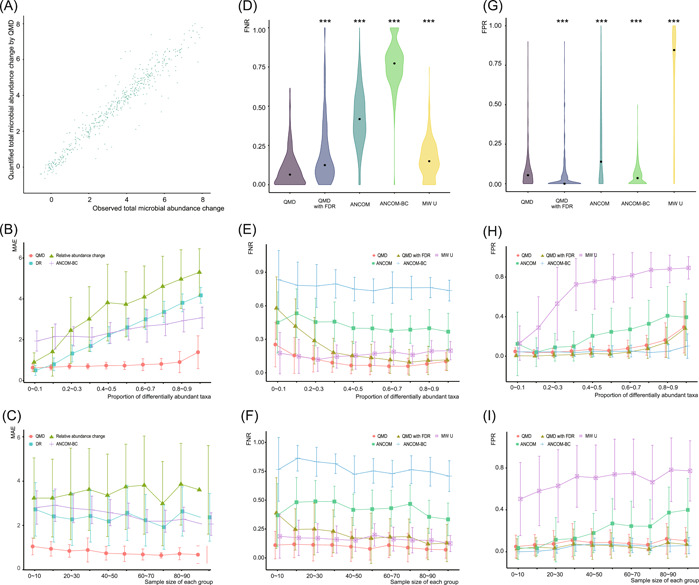
Benchmark simulation result of H2029 series. (A) Quantified total microbial abundance changes by QMD are linearly correlated with the observed total microbial abundance changes, indicating QMD is a qualified tool for total microbial abundance change estimation. (B) and (C) Proportion of differentially abundant taxa and sample size of each group affect the MAE of QMD, DR, and ANCOM‐BC. (D) FNR of QMD, QMD with FDR adjustment, ANCOM, ANCOM‐BC in differentially abundant taxa identification. (E) and (F) The proportion of differentially abundant taxa and sample size of each group affect the FNR of each method. (G) FPR of QMD, QMD with FDR adjustment, ANCOM, ANCOM‐BC in differentially abundant taxa identification. (H)–(I), Proportion of differentially abundant taxa and sample size of each group affect the FPR of each method. ANCOM, Analysis of the composition of microbiomes; ANCOM‐BC, analysis of compositions of microbiomes with bias correction; DR, differential ranking; FNR, false negative rate; FPR, false positive rate; MAE, mean absolute error; QMD, quantification of microbial absolute abundance differences.

Linear regressions were performed on the estimated total microbial abundance change $\hat{∆\Psi }$ by QMD and the observed ∆Ψ. *R* ^2^ (adj) were greater than 0.97 in all three simulation series (Figure 3A, Supporting Information: Figures S4A and S5A, and Table 2). This implies that QMD is a tool qualified for ∆Ψ estimation.

**Table 2:** Linear regression of QMD‐quantified total microbial abundance and the truly observed quantified total microbial abundance

Data set series	Coefficients	Multiple *R* ^2^	Adjusted *R* ^2^	Degrees of freedom	*F*‐statistic	*p*‐value
H2029	0.992488	0.9779	0.9779	499	2.209e + 04	<2.2e − 16
Obesity	0.995639	0.9786	0.9786	497	2.272e + 04	<2.2e − 16
GP	0.999599	0.9972	0.9972	499	1.787e + 05	<2.2e − 16

Only a few methods can estimate absolute abundance differences. We compared ANCOM‐BC, DR, QMD, and relative abundance change (RAC). The RAC method directly uses logged relative abundance differences between groups as absolute abundance differences. The median of mean absolute error (MAE) of QMD was 0.69 for H2029, 0.77 for Obesity, and 0.01 for GP. Obviously, QMD outperformed the other three methods in quantifying abundance changes (see Figure 3B,C, Supporting Information: Figures S4B,C and S5B,C, and Table 3).

**Table 3:** MAE median of absolute abundance differences quantification using ANCOM‐BC, DR, QMD, and RAC in benchmark simulation

Methods	H2029	Obesity	GP
QMD	0.69	0.77	0.01
RAC	3.59	3.88	4.99
DR	2.35	2.35	2.39
ANCOM‐BC	2.42	2.57	0.16

Abbreviations: ANCOM‐BC, analysis of compositions of microbiomes with bias correction; DR, differential ranking; QMD, quantification of microbial absolute abundance differences; RAC, relative abundance change.

QMD was built on the assumption that most taxa undergo relatively small absolute abundance changes. As expected, the proportion of differentially abundant taxa affected the estimation accuracy in this study. The MAE increased with increasing proportion. The larger the proportion of differentially abundant taxa, the more the abundance would be perturbed. QMD is more robust when confronted with large proportions than other methods. The median MAE of QMD stayed below 1 until the proportion increased to 90%. DR achieved a small MAE median (lower than 1) when the proportion of differentially abundant taxa was less than 20%. When the proportion of differentially abundant taxa was less than 10%, DR performed better than QMD (Figure 3B, Supporting Information: Figure S4B and S5B).

Conversely, large sample size can increase statistical inference power. The MAE decreased slightly with increasing sample sizes for the four methods except for RAC. For example, the median MAE for QMD was 0.98 for the simulated instance H2029 with a sample size of less than 10. This value was improved to 0.64 with a sample size of about 90–100 (Figure 3C, Supporting Information: Figures S4C and S5C).

### QMD balances false negative rate (FNR) and false positive rate (FPR) well in differentially abundant taxa identification

Next, FPR and FNR were used to evaluate and compare the differentially abundant taxa identification power of QMD, QMD with FDR adjustment, ANCOM, ANCOM‐BC, and MWU in the simulation. The MWU method applies the Mann–Whitney *U* test directly to the logged relative abundance. DR was not included because it does not provide a *p* value or a threshold to filter differentially abundant taxa.

The simulation revealed that ANCOM‐BC had a seriously inflated FNR and that MWU had a seriously inflated FPR. These two methods did not maintain a good balance between the FNR and FPR control. QMD and QMD with FDR adjustment performed better than ANCOM in both FNR and FPR. The medians of FPR and FNR were higher in ANCOM (Figure 3D,G, Supporting Information: Figures S4D,G and S5D,G, and Tables 4 and 5).

**Table 4:** FPR median of ANCOM‐BC, ANCOM, QMD, QMD with FDR adjustment, and MWU in benchmark simulation

Methods	H2029	Obesity	GP
QMD	0.05	0.05	0.15
QMD with FDR adjustment	0	0	0.11
ANCOM	0.14	0.08	0.08
ANCOM‐BC	0.04	0	0
MWU	0.85	0.83	1

Abbreviations: ANCOM, Analysis of the composition of microbiomes; ANCOM‐BC, analysis of compositions of microbiomes with bias correction; FDR, false discovery rate; MWU, Mann–Whiney *U* test.

**Table 5:** FNR median of ANCOM‐BC, ANCOM, QMD, QMD with FDR adjustment, and MWU in benchmark simulation

Methods	H2029	Obesity	GP
QMD	0.06	0.08	0
QMD with FDR adjustment	0.12	0.15	0
ANCOM	0.42	0.47	0
ANCOM‐BC	0.77	0.85	0.07
MWU	0.15	0.17	0

Abbreviations: ANCOM, Analysis of the composition of microbiomes; ANCOM‐BC, analysis of compositions of microbiomes with bias correction; FDR, false discovery rate; MWU, Mann–Whiney U test.

QMD with FDR performed better in FPR than QMD alone (Figure 3H,I, Supporting Information: Figures S4H,I and S5H,I, and Table 4). In the case of small differentially abundant taxa proportion or small sample size, the FNR is larger after FDR adjustment (Figure 3E,F, Supporting Information: Figures S4E,F and S5E,F, and Table 5). In summary, we recommend an FDR adjustment for QMD when using QMD for a sample size larger than 50. If the sample size is smaller, it is not necessary to turn the FDR adjustment on.

## DISCUSSION

Differentially abundant taxa can help us to reveal changes in the microbiome between groups. As a result, their identification has become a common aspect of microbiome analysis, but identifying differentially abundant taxa is particularly challenging [3]. The deviation between relative abundance differences and absolute abundance differences may be greatly misleading regarding the identification of differentially abundant taxa, producing conflicting results. In this study, we developed and validated a simple, user‐friendly QMD method to quantify absolute abundance differences between groups for each taxon.

QMD reveals that the deviation is equal to the total microbial absolute abundance change ∆Ψ between groups. Through calculating this deviation, QMD quantifies the absolute abundance differences between groups of each taxon as its relative abundance change plus the deviation. QMD deals with only one unknown variable: the total change in absolute abundance ∆Ψ. This feature has greatly reduced the QMD model's complexity and made its analysis more robust in comparison with previous methods (e.g., DR), which estimate unknown changes in absolute abundance for hundreds of taxa at a time. The benchmark simulation conducted in this study corroborated this conclusion. QMD estimated microbial absolute abundance differences more accurately than DR and ANCOM‐BC. DR's performance quickly deteriorated with an increasing proportion of differentially abundant taxa. As stated by its authors, the ANCOM‐BC method performs poorly with very small sample sizes [1].

The changes in microbial abundance of each taxon estimated by QMD create a quantitative description of microbiome dynamics between groups. The p‐value and FDR‐adjusted *q* value in the QMD are provided to identify the statistically significant differentially abundant taxa. The benchmark simulation results show that QMD controls the FNR and FPR well, even in small samples or with a large proportion of differentially abundant taxa.

In our study, we found that the relative abundance changes are linearly correlated to absolute microbial abundance changes, and the top abundance increased or decreased taxon in relative abundance changes is identical to that in absolute microbial abundance changes. DR concluded a similar finding that the ranks of relative abundance changes are identical to the ranks of absolute microbial abundance changes [2]. In this regard, DA identification based on relative abundance is comparable to QMD if researchers only focus on the top changed taxa. On the other hand, for these differentially abundant taxa not in the largest‐changed taxon set, it is necessary to take the total microbial abundance change into account. As shown in Supporting Information: Figure S3, the total microbial abundance change might bring out an opposite trend in abundance changing. Inflated zeros and overdispersion of microbiome abundance should be taken into consideration during DA identification. QMD removed the zero count of taxa and pseudocount was not embedded to avoid potentially biased results [23]. Integrating other overdispersion modeling might be a direction for the future development of the QMD model.

Given the mathematical property of relative abundance data, if a common decrease or increase occurs in all taxa's abundance, the QMD will not function well. In the extreme case, treatment with broad‐spectrum antibiotics might cause a common decrease in all taxa's microbial absolute abundance. In this case, formula 9 will degenerate to ${∆M}_{i}=∆\Psi$, and the relative abundance will not change between groups.

## METHODS

### Simulation framework for performance evaluation

We modified McMurdie et al. protocol and built the simulation benchmark [24]. Supporting Information: Figure S6 describes the simulation framework, which is based on a real data shuffling strategy. Three data sets, GP, H2029, and Obesity, were selected as the original pool. The GP data set [25] was chosen because it has already been used in several studies in the DA‐related literature [1, 7, 24]. The other two databases contain the gut microbiome data of a healthy population aged 20–29 years and an obese population, respectively, from GMrepo [26].

Since $a_{i,j}^{K}=\Psi_{j}^{K}\times o_{i,j}^{K}$ (see Supporting Information File), a uniformly distributed $\Psi_{j}^{K}$ was designated for each sample to aid in the generation of artificial absolute abundance data. We obtained the preliminary simulation instances by resampling from the artificial absolute abundance data pool. The resampling sample size follows the uniform distribution U(6,100).

For each simulation instance, the proportion of differentially abundant taxa was randomly set following the uniform distribution U(5%,95%). Then, a random subset of taxa was chosen according to the proportion. Microbial abundance changes were adapted to these taxa in the treatment group. The simulated change in absolute abundance for each taxon follows the uniform distribution U(−10,10).

We obtained 1500 simulation instances for the series of GP, H2029, and Obesity, with 500 instances each. The simulation conditions cover a random combination of sample sizes U(6,100), proportions of differentially abundant taxa U(5%,95%), and absolute abundance change U(−10,10) (Supporting Information: Figure S7).

In the evaluation stages, two types of performance were measured. In the absolute abundance differences quantification part, the MAE (Mean Absolute Error) was used to assess the quantification accuracy of ANCOM‐BC, DR, QMD, and RAC. It is calculated as follows:

(13) $$\mathrm{MAE}=\frac{1}{n}\sum_{i}|\hat{∆M_{i}}-∆M_{i}|$$

where $\hat{{∆M}_{i}}$ is the quantified absolute abundance differences for ${\mathrm{Taxa}}_{i}$.

FNR and FPR were used to assess the differentially abundant taxa identification power of QMD, QMD with FDR adjustment, ANCOM, ANCOM‐BC, and MWU. Perturbed taxa are considered differentially abundant between groups. Taxa that had not been perturbed and identified as DA were considered false positives. Taxa that had been perturbed but not identified as DA were considered false negatives. The following decision table to calculate the FNR and FPR. The *Positive by QMD model, Negative by QMD model* data were derived from the QMD model by statistical test and *the Positive at Real data, Negative at Real data* were derived from the simulation parameter set to calculate FNR and FPR (Table 6).

**Table 6:** decision table to calculate the FNR and FPR

	Positive by QMD model	Negative by QMD model
Positive at real data	True positive	False negative (type II error)
Negative at real data	False positive (type I error)	True negative

For correctness validation on real data, we selected datasets from the literature [14] and [13] which measured the absolute microbial abundance experimentally. Barlow et al. provided the absolute abundance data for each taxon. From absolute abundance data, the relative abundance data were calculated following Formula (7). Vandeputte et al. provided the total taxa abundance of each sample counted by flow cytometry. The relative abundance of Vandeputte et al. was derived from 16s rRNA sequencing and the absolute abundance of the taxon was derived by the total taxa abundance multiplied by its relative abundance. Given both the absolute and relative abundance data, the FNR (type II error) and FPR (type I error) were calculated to validate the QMD model.

In the simulation ANCOM‐BC (https://github.com/FrederickHuangLin/ANCOM-BC-Code-Archive/blob/master/scripts/ancom_bc.R), ANCOM (https://github.com/mortonjt/ancomP), DR (the DR algorithm were extracted from https://github.com/knightlab-analyses/reference-frames/blob/master/ipynb/simulation-benchmark.ipynb) were run at the default parameter sets. In ANCOM‐BC, the program discarded taxa with zeros proportion ≥ 0.9 and library size cut threshold lib.cut was set to 1000, using Bonferroni p‐value adj method, tol.EM = 1e−5; max.iterNum = 100; perNum = 1000; alpha = 0.05). Permutative‐ANOVA was used in ANCOM analysis, and permutations = 1000. In DR, batch_size = 3, learning_rate = 1e−3, beta_scale = 1.

It has been reported pseudocount might lead to biased results [23]. In QMD, we do not use pseudocount because we cannot determine whether the zero count is brought out by insufficient sequence depth or just because the taxon couldnot colonize. Instead, QMD provides two ways to handle the filtered‐out taxon, the first is to exclude these taxa from the next step analysis directly, and the second way is to sum these taxon abundances as the filtered‐out‐taxa to enter the next step analysis as other normal taxa. In the simulation, QMD discarded taxon detected in less than five samples, Benjamini/Hochberg for independent tests were used for FDR correction, and permutations were set to 500. The MWU method applies the Mann‐Whitney U test directly to the logged relative abundance. The RAC method directly uses logged relative abundance differences between groups as absolute abundance differences. For all methods, 0.05 were used to filter significantly changed taxa.

### Analysis of 16S rRNA sequences

To validate the correctness of QMD, raw 16S rRNA sequencing data for B1 and B2 were obtained from the European Nucleotide Archive, with accession codes PRJEB21504 and ERP023761 [13]. Feature tables were created with QIIME 2 [6]. Taxonomy was assigned to ASVs using the SILVA naive Bayes taxonomy classifier [27, 28].

To compare the total microbial abundance change pattern among different human diseases, the genus level abundances of PRJEB7949, PRJNA368966, PRJNA385949, PRJNA388210, PRJNA389280, PRJEB6070, PRJNA445640 were obtained from GMrepo [26].

For PRJNA763023, and PRJNA433269, the unique 16s reads and feature tables of samples were extracted by vsearch v2.17.1_linux_x86_64 [29].

## AUTHOR CONTRIBUTIONS

Xingyin Liu and Kai Mi conceived and designed this study. Kai Mi developed the methodology and constructed the online service and local packages. Kai Mi performed the statistical analysis. Kai Mi and Xingyin Liu prepared and wrote the manuscript. Yuyu Xu and Yiqing Li provided advice on analyses and the interpretation of results. Kai Mi and Yiqing Li prepared the tutorial video. All authors read, checked, and approved the final manuscript.

## CONFLICT OF INTEREST

The authors declare no conflicts of interest.

## Supporting information

Supporting information.

Supporting information.

## Data Availability

All data generated in the correctness validation and benchmark simulation, and codes developed by the authors are available at https://github.com/Xingyinliu-Lab/QMD. A graphical user interface version and a character user interface version of the QMD software are also provided at https://github.com/Xingyinliu-Lab/QMD under GPL licence. Supporting Information materials (figures, tables, scripts, graphical abstract, slides, videos, Chinese translated version, and update materials) may be found in the online DOI or iMeta Science http://www.imeta.science/.
