# Supplementary material for: QMD: A new method to quantify microbial absolute abundance differences between groups
Source: Imeta. 2023 Jan 5;2(1):e78. doi: 10.1002/imt2.78 (PMC10989753; doi:10.1002/imt2.78)
Supplement: Supplementary file 1 — Supporting information. [file IMT2-2-e78-s001.docx]

**Figure S1.** **QMD validation in STOOL dataset.**

(A) Validation of the relationship of relative abundance changes and the absolute abundance changes. (B) The visualization of traversal processes to find the quantified absolute abundance differences by QMD in STOOL. A magnifier was provided in the optimal target zone. (C) Performance comparison of QMD, DR and ANCOM-BC in absolute abundance differences quantification.

**Figure S2. Performance comparison in B1 and B2 validation datasets.**

(A) Performance comparison of QMD, DR and ANCOM-BC in absolute abundance differences quantification in B1 dataset. (B) Performance comparison of QMD, DR and ANCOM-BC in absolute abundance differences quantification in B2 dataset.

**Figure S3. Adopting QMD to human gut microbiome.**

(A) The total absolute abundance changes between diseased and health gut microbiome across different human diseases. (B) The absolute abundance changes in colorectal cancer with comparison to the relative abundance changes.

**Figure S4. Benchmark simulation result of Obesity series.**

(A) Quantified total microbial abundance changes by QMD are linearly correlated with the observed total microbial abundance changes, indicating QMD is a qualified tool for total microbial abundance change estimation. (B)-(C), Proportion of differentially abundant taxa and sample size of each group affect the MAE of QMD, DR and ANCOM-BC. (D) FNR of QMD, QMD with FDR adjustment, ANCOM, ANCOM-BC in differentially abundant taxa identification. (E)-(F) Proportion of differentially abundant taxa and sample size of each group affect the FNR of each method. (G) FPR of QMD, QMD with FDR adjustment, ANCOM, ANCOM-BC in differentially abundant taxa identification. (H)-(I) Proportion of differentially abundant taxa and sample size of each group affect the FPR of each method.

**Figure S5. Benchmark simulation result of GP series.**

(A) Quantified total microbial abundance changes by QMD are linearly correlated with the observed total microbial abundance changes, indicating QMD is a qualified tool for total microbial abundance change estimation. (B)-(C), Proportion of differentially abundant taxa and sample size of each group affect the MAE of QMD, DR and ANCOM-BC. (D) FNR of QMD, QMD with FDR adjustment, ANCOM, ANCOM-BC in differentially abundant taxa identification. (E)-(F) Proportion of differentially abundant taxa and sample size of each group affect the FNR of each method. (G) FPR of QMD, QMD with FDR adjustment, ANCOM, ANCOM-BC in differentially abundant taxa identification. (H)-(I) Proportion of differentially abundant taxa and sample size of each group affect the FPR of each method.

**
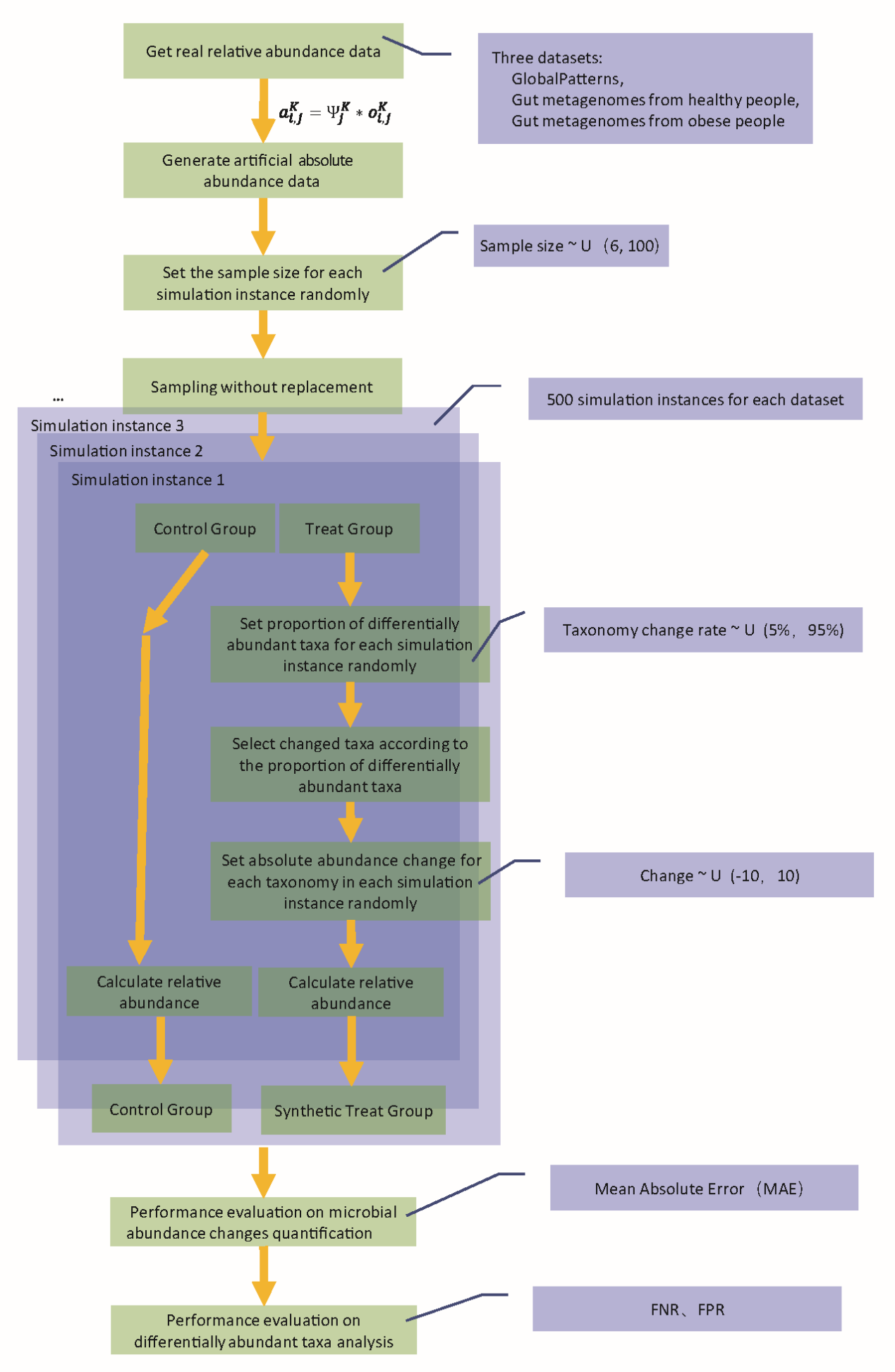
**

**Figure S6. Benchmark simulation framework.**

Totally 1500 simulation instances were built, 500 for each dataset series, i.e. H2029, Obesity, GP.

**Figure S7. Randomized simulation conditions.**

The Proportion of differentially abundant taxa, the sample size and the changes for every taxon are randomized in the simulation. (A)-(D), Spatial distribution and 2-D distribution of the three randomized factors in H2029 series. (E)-(H), Spatial distribution and 2-D distribution of the three randomized factors in Obesity series. (I)-(L), Spatial distribution and 2-D distribution of the three randomized factors in GP series. Note average absolute abundance change is the mean of changes of all taxa in a simulation instance. Thus, the variance of average absolute abundance change is decreasing with increasing proportion of differentially abundant taxa.
